# Identification and immune landscape analysis of fatty acid metabolism genes related subtypes of gastric cancer

**DOI:** 10.1038/s41598-023-47631-6

**Published:** 2023-11-22

**Authors:** Rong Huang, Tai-Liang Lu, Rui Zhou

**Affiliations:** 1 Department of Laboratory, Hexian Memorial Hospital of Panyu District, No. 2, Qinghe East Road, Panyu District, Guangzhou, 511400 China; 2 Department of Gastrointestinal Surgery, Hunan Provincial People’s Hospital, The First Affiliated Hospital of Hunan Normal University, Changsha, 410005 China

**Keywords:** Biotechnology, Gastroenterology

## Abstract

Fatty acid metabolism (FAM) is associated with prognosis and immune microenvironment remodeling in many tumors. It is currently unknown how FAM affects the immunological microenvironment and prognosis of Gastric cancer (GC). Therefore, the current work aims to categorize GC samples based on the expression status of genes involved in FAM and to identify populations that might benefit from immunotherapy. In total, 50 FAM genes associated with overall survival (OS) were determined through univariate Cox proportional hazard regression analysis by mining the public TCGA and GEO databases. The GSE84437 and TCGA-STAD cohort samples were divided into two clusters using the "NMF" R package. According to the survival curve, patients in Cluster-1 showed considerably longer OS than those in Cluster-2. Patients in Cluster-1 exhibited earlier T stages, more intestinal GCs, and were older. MSI molecular subtypes were mainly distributed in Cluster-1, while GS molecular subtypes were distributed primarily in Cluster-2. There were 227 upregulated and 22 down-regulated genes (logFC > 1 or logFC < − 1, FDR < 0.05) in Cluster-2 compared with Cluster-1. One hub module (edges = 64, nodes = 12) was identified with a module score of 11.636 through Cytoscape plug-in MCODE. KEGG and GO analysis showed that the hub genes were associated with the cell cycle and cell division. Different immune cell infiltrates profile, and immune pathway enrichment existed between the subtypes. In conclusion, the current findings showed that practically all immunological checkpoint and immunoregulatory genes were elevated in patients with Cluster-2 GC, indicating that FAM subtypes may be crucial in GC immunotherapy.

## Introduction

Gastric cancer (GC) is one of the most common malignant tumors of the digestive tract. Globally, GC ranks fifth in incidence and fourth in mortality, according to Global Cancer Statistics 2020^1^. In 2020, 770,000 deaths (65% men) were reported worldwide due to GC. GC is among the top primary causes of cancer deaths in 42 countries and the leading cause in 13 countries. Eastern Asia was home to 56.6% of all deaths worldwide, 48.6% of which were in China alone. Male mortality rates varied by area, from under 5 per 100,000 in New Zealand/Australia, Northern Europe, and Northern America to over 20 per 100,000 in Eastern Asia. Tajikistan (15.4) and Mongolia (36.5 per 100,000) had the highest male and female mortality rates, respectively^1^. The strategy for treating GC is a comprehensive treatment based on surgery. Although with the popularization of gastroscopy, the proportion of early GC increases year by year^2^, and with the promotion of precise medical concepts and the development of surgical technology and instruments, the prognosis of GC patients has been improved^3^, the overall prognosis is still not ideal. Classification of GC into molecular subtypes and metabolic subtypes suitable for precision therapy may further improve the prognosis of GC^4^.

A carbon, hydrogen, and oxygen compound forms fatty acids (FA). It is the primary constituent of glycolipid, phospholipid, and neutral fat. It is also the main substance for cell energy supply. Fatty acid metabolism (FAM) includes catabolism and anabolism. Under oxygen, FA can oxidize and break down, producing energy known as fatty acid oxidation (FAO). Acetyl-CoA, a byproduct of glycolysis, the tricarboxylic acid cycle, and amino acid breakdown is used in fatty acid synthesis (FAS) to create 16-carbon intermediates and transform them into different fatty acids. The importance of FAM in cancer progression, survival, and metastasis has received increasing attention in recent years^5–7^.

According to studies, lipid metabolism assists in helping cancer cells proliferate rapidly, survive, migrate, invade, and metastasize. Additionally, increased lipid production or uptake aids in the growth of cancer cells and accelerates the development of tumors^8^. Lipid metabolism changes are important metabolic phenotypes of cancer cells^9^. Therefore, blocking lipid supply in cancer cells has significant implications for cell bioenergetics, membrane biosynthesis, and intracellular signaling processes^10^. Studies have shown that the gene set related to FAM can distinguish the clinicopathological features of glioma, and there is a potential association between FAM and the immunophenotype of glioma^11^. However, studies on the characteristics and prognosis of GC subtypes based on genes related to FAM are still limited^12^.

The emergence of immunotherapy has brought a glimmer of hope to treating patients with advanced GC, but there are also some difficulties, such as unsatisfactory screening population^13^. Specific populations with GC benefit from PD-1 antibodies. Currently, patients with high microsatellite instability (MSI-H) and Epstein-Barr Virus (EBV) positive have unique characteristics, and the efficacy and benefits of immunotherapy are relatively clear^14^. However, MSI-H and EBV-positive patients account for only about 7% to 8% of the total population with advanced GC, and there is no clear and effective screening method for most of the rest of the population^15,16^.

Many studies have shown that FAM affects the main immune cells in tumor microenvironment^17,18^. Therefore, we hypothesized that classifying GC samples based on the expression status of FAM genes might further screen out the population that may benefit from immunotherapy.

This study used the GSE84437 data set to screen out prognostic fatty acid metabolization-related gene sets. We also used this gene set to perform non-negative matrix factorization (NMF) consensus clustering in GC patients to identify fatty acid metabolization-related subtypes. TCGA-STAD data sets were used to verify the conservatism of subtypes. Then, we compared the differences in prognosis and immune microenvironment characteristics among different subtypes, analyzed the clinicopathological features associated with subtypes, and evaluated the FAM subtypes as independent prognostic factors of GC. We also examined the relationship between FAM subtypes^19^, the expression of immunological checkpoint and immune regulatory genes, and the molecular properties of FAM subtypes.

## Results

### Identification of FAM genes associated with prognosis in GC

The publicly available microarray dataset GSE84437, composed of 433 GC samples, was downloaded from GEO along with the complete clinicopathological and follow-up data. 309 out of 332 FAM genes had expression data in this dataset. 50 FAM genes related to OS were picked out by univariate Cox proportional hazard regression (Supplementary Table S2).

### Identification of FAM-related subtypes in GC and analysis of the prognosis value of the subtypes

**Figure 1:**
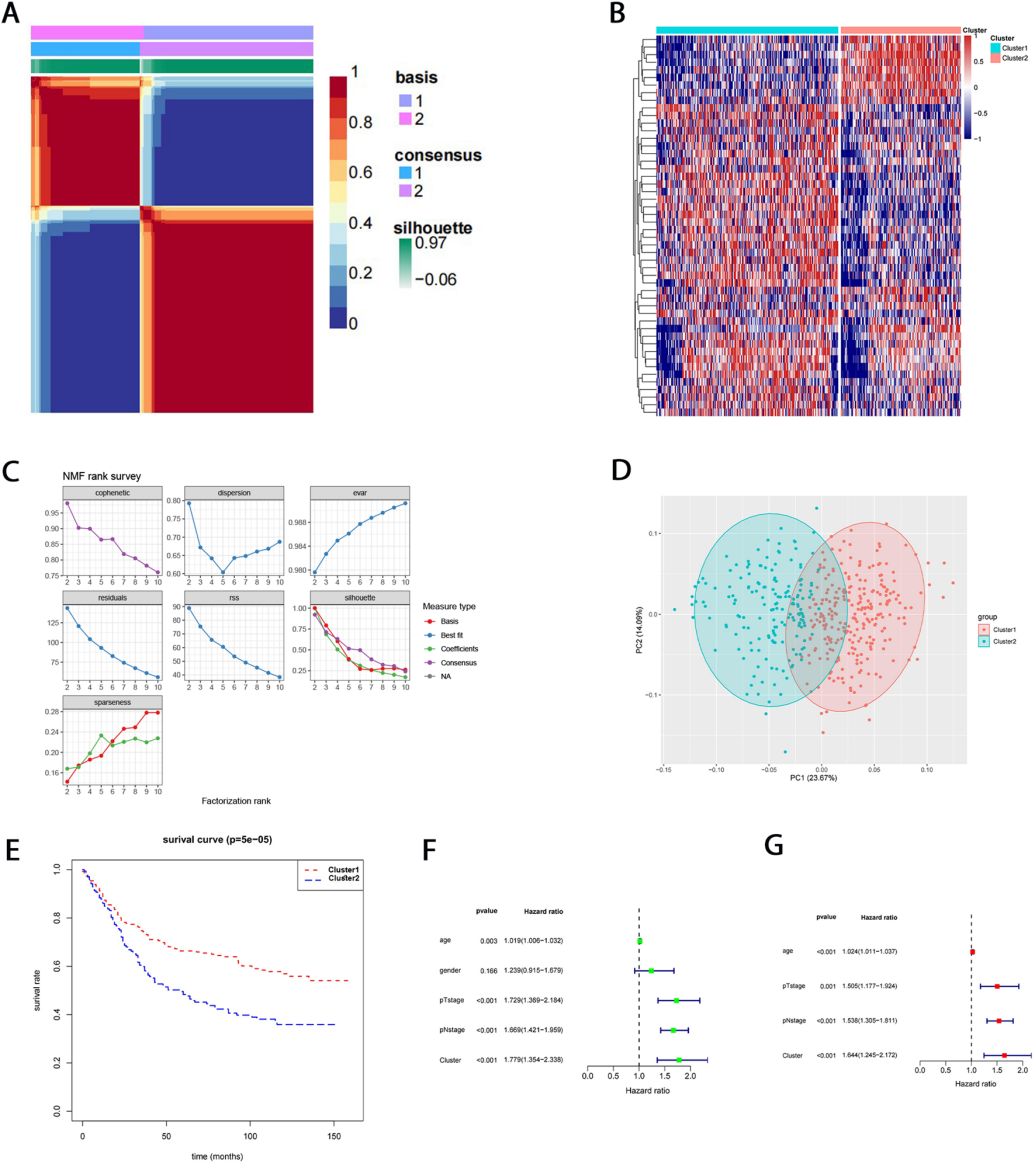
Identification of FAM-related subtypes in GC and analysis of the prognosis value of the subtypes. (**A**) The consensus map of NMF clustering results in the GSE84437 dataset with rank k = 2. (**B**) Heatmap of prognostic FAM gene expression. (**C**) The trend of the cophenetic, dispersion, evar, residuals, rss, silhouette, and sparseness coefficients at different ranks k. (**D**) The principal component analysis results for GSE84437 cohort samples. (**E**) Kaplan–Meier survival curves of overall survival in Cluster-1 and Cluster-2 GC patients. (**F**) Forest plot of GSE84437 cohort univariable Cox regression analysis. (**G**) Forest plot of GSE84437 cohort multivariable Cox regression analysis.

Based on 50 survival-related FAM gene expressions, we divided the 433 GC samples into two subtypes through NMF consensus clustering. For any desired rank k, the NMF algorithm groups the samples into k clusters. To find out the robust rank k, we performed the NMF with rank k from 2 to 10 (Fig. 1A, Supplementary Fig. S1). According to the cophenetic coefficient and silhouette score, the stable and strong k was 2 (Fig. 1B). Cluster-1 included 261 GC samples, and Cluster-2 comprised 172 GC samples. The heatmap showed that Cluster-1 and Cluster-2 exhibited distinct gene expression profiles (Fig. 1C). PCA analysis was used to confirm the distribution patterns of the clusters (Fig. 1D).

To explore the clinical significance of classification, we performed the survival analysis, and the result showed that the GC patients of Cluster-1 have a better prognosis than that of Cluster-2 (p = 5e−05) (Fig. 1E). The Univariate Cox regression analysis and multivariate Cox regression analysis showed that the FAM-related subtypes were independent prognostic factors (Fig. 1F, G). We used the TCGA STAD cohort as a validation cohort to verify the stability and robustness of our clustering. The findings demonstrated that the TCGA STAD cohort was also robustly categorized into two clusters (Supplementary Fig. S2, S3) and that the PCA analysis divided these two clusters (Supplementary Fig. S4). Additionally, the findings revealed that GC patients in Cluster-1 had a better prognosis than those in Cluster-2 (p = 0.00522) (Supplementary Fig. S5, S6, and S7).

### Clinicopathological features of FAM-related subtypes

**Figure 2:**
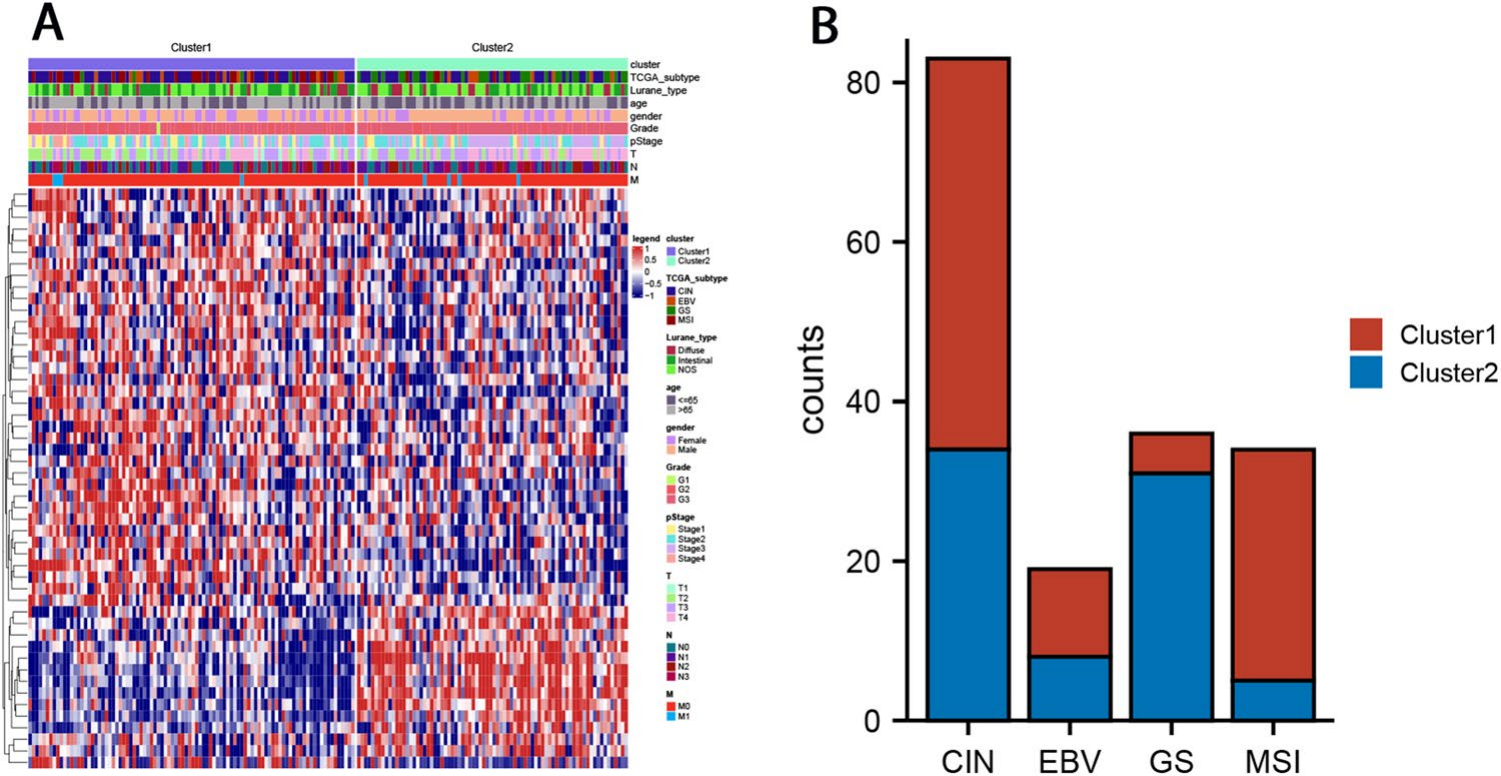
Clinicopathological features of FAM-related subtypes. (**A**) Heatmap of clinical features of FAM subtypes. (**B**) Composition of TCGA molecular subtypes in FAM subtypes.

Since the TCGA-STAD cohort has relatively complete clinicopathological data, we used the data of this cohort to explore the differences in clinicopathological characteristics among GC FAM subtypes. As shown in Table 1 and Fig. 2A, compared with Cluster-2, Cluster-1 patients were older, and the proportion of intestinal-type GC and T1/T2 stage GC were higher. However, the two groups had no significant differences in gender, pathological stage, presence or absence of lymph node metastasis, and distant metastasis. At the same time, considering that gastric-esophageal junction carcinoma has unique characteristics, we further analyzed whether there are differences in the distribution of gastric-esophageal junction carcinoma and GC between Cluster-1 and Cluster-2. The results showed no statistically significant difference between the two groups in gastroesophageal junction carcinoma and GC distribution.

**Table 1:** Clinicopathological features of FAM-related subtypes of the TCGA-STAD cohort.

	Cluster-1 (n = 193)	Cluster-2 (n = 156)	*χ*^2^	*P*
Age				
≤ 65	76	77	30.298	≤ 0.001
> 65	117	29		
Gender				
Male	125	99	0.064	0.8
Female	68	57		
Lurane_type				
Intestinal	102	53	9.24	0.002
Diffuse	31	39		
Grade				
G1	5	3	15.827	< 0.001
G2	87	39		
G3	101	114		
PStage				
Stage I + II	99	65	3.211	0.073
Stage III + IV	94	91		
T				
T1 + T2	61	29	7.638	0.006
T3 + T4	132	127		
N				
N0	65	44	1.203	0.273
N+	128	112		
M				
M0	182	144	0.557	0.456
M1	11	12		
Anatomic_subdivision				
EGJ	23	16	0.3	0.584
Stomach	163	137

TCGA database divided GC patients into four molecular subtypes, namely chromosomal instability (CIN), EBV, genomically stable (GS), and MSI, each with unique clinical characteristics. We also explored the relationship between FAM-related subtypes and TCGA GC molecular subtypes. The results showed little difference in the distribution of CIN and EBV between the two groups, but MSI was mainly distributed in Cluster-1 GC patients. In contrast, GS was distributed primarily among Cluster-2 GC patients (Table 2, Fig. 2B).

**Table 2:** The relationship between FAM-related subtypes and TCGA GC molecular subtypes.

TCGA_subtype	Cluster-1 (n = 94)	Cluster-2 (n = 78)	*χ*^2^	*P*
CIN	49	34	37.742	< 0.001
EBV	11	8		
GS	5	31		
MSI	29	5		

### Differential expression gene (DEG) analysis, PPI, and Functional enrichment analysis of FAM-related subtypes

**Figure 3:**
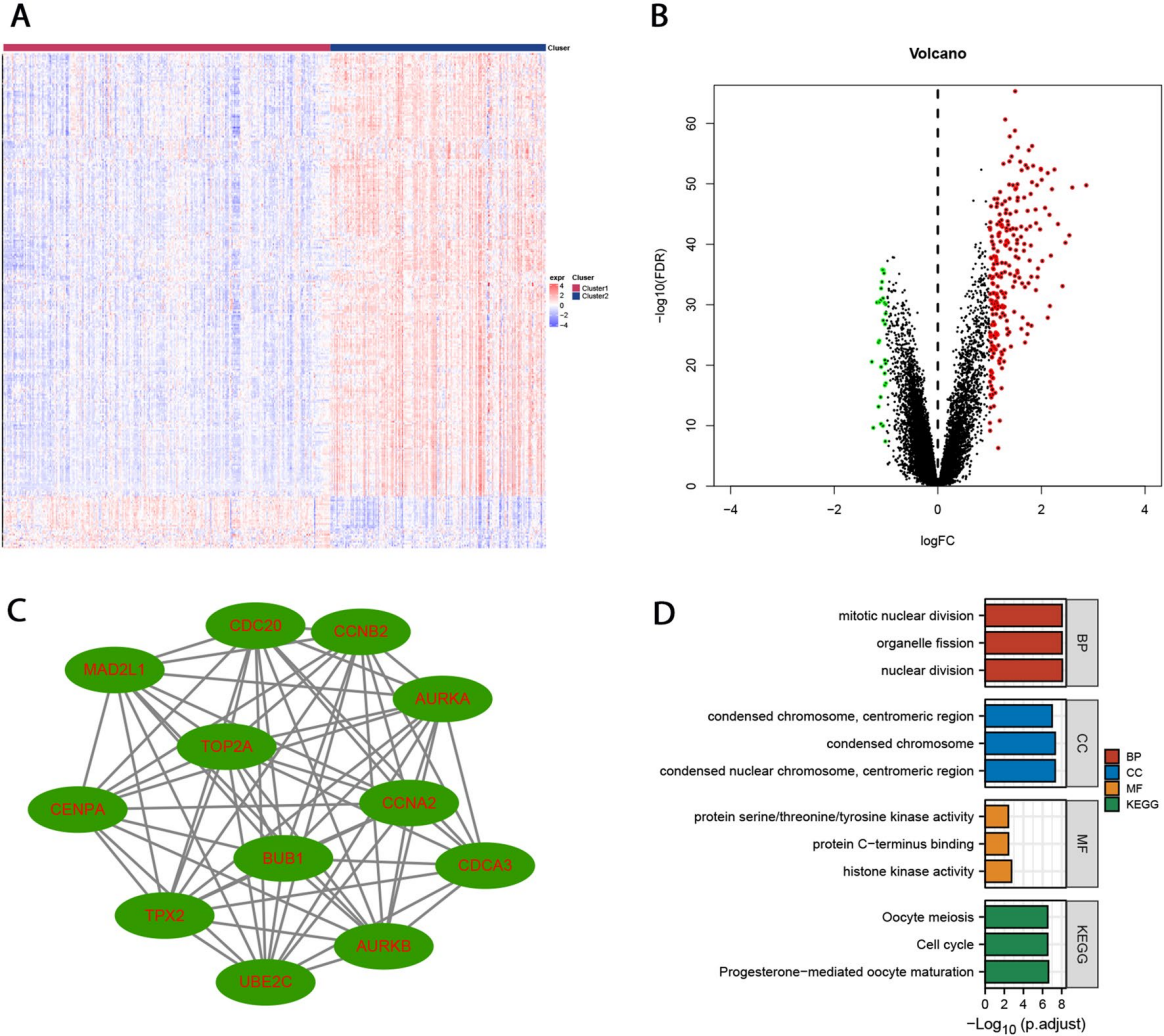
Differential expression gene(DEG) analysis, PPI, and Functional enrichment analysis of FAM-related subtypes. (**A**) Heatmap of differentially expressed genes of FAM subtypes. (**B**) Volcano map of differentially expressed genes of FAM subtypes. (**C**) Cytoscape plug-in MCODE identified one hub module (edges = 64, nodes = 12) with a module score 11.636. (**D**) GO and KEGG functional enrichment analysis of the hub module genes.

DEG analysis, PPI analysis, and GO and KEGG functional enrichment analysis were performed to explore the characteristic molecular characteristics between subtypes. Differential expression analysis showed 227 upregulated genes and 22 down-regulated genes (logFC > 1 or logFC < −1, FDR < 0.05) in the Cluster-2 subtype compared with the Cluster-1 subtype (Fig. 3A, B and Supplementary Table S3). String database was used to analyze the PPI of differentially expressed genes, and Cytoscape plug-in MCODE was used to predict the hub module. The threshold value was set as MCODE score ≥ 4 and node ≥ 6. One hub module (edges = 64, nodes = 12) was identified with a module score of 11.636. The nodes included AURKB, TPX2, BUB1, UBE2C, CDCA3, CCNA2, AURKA, CCNB2, TOP2A, MAD2L1, CENPA, and CDC20 (Fig. 3C). GO and KEGG functional enrichment analysis were performed on the hub module genes (Fig. 3D).

The results show that the most significant enrichment analysis of biological processes is a nuclear division (GO:0000280), organelle fission (GO:0048285), and mitotic nuclear division (GO:0140014) (Table 3). The most significant results of cell component enrichment analysis were: Condensed nuclear idea, Centromeric region (GO:0000780), Condensed idea (GO:0000793), Condensed idea, Centromeric Region (GO:0000779) (Table 3). The most significant results of molecular function enrichment analysis are Histone kinase activity (GO:0035173), protein C-terminus binding (GO:0008022), histone kinase activity (GO:0035173), Protein C-terminus binding (GO:0008022) Protein serine/threonine/tyrosine kinase activity (GO: 0004712) (Table 3). The most significant KEGG enrichment analysis was Progesterone mediated oocyte maturation (HSA04914), Cell cycle (HSA04110), and oocyte meiosis (HSA04114) (Table 3).

**Table 3:** GO and KEGG functional enrichment analysis of the hub module genes.

Ontology	ID	Description	Gene ratio	Bg ratio	p-value	p.adjust	q-value	Gene ID	Count
BP	GO:0000280	Nuclear division	8/12	407/18,670	2.19e−11	8.63e−09	3.20e−09	BUB1/CDC20/MAD2L1/AURKA/TOP2A/AURKB/UBE2C/TPX2	8
BP	GO:0048285	Organelle fission	8/12	449/18,670	4.79e−11	9.37e−09	3.47e−09	BUB1/CDC20/MAD2L1/AURKA/TOP2A/AURKB/UBE2C/TPX2	8
BP	GO:0140014	Mitotic nuclear division	7/12	264/18,670	7.79e−11	9.37e−09	3.47e−09	BUB1/CDC20/MAD2L1/AURKA/AURKB/UBE2C/TPX2	7
CC	GO:0000780	Condensed nuclear chromosome, Centromeric region	4/12	26/19,717	1.17e−09	4.78e−08	7.19e−09	BUB1/CENPA/AURKA/AURKB	4
CC	GO:0000793	Condensed chromosome	6/12	223/19,717	1.71e−09	4.78e−08	7.19e−09	BUB1/CENPA/MAD2L1/AURKA/TOP2A/AURKB	6
CC	GO:0000779	Condensed chromosome, centromeric region	5/12	118/19,717	5.40e−09	1.01e−07	1.52e−08	BUB1/CENPA/MAD2L1/AURKA/AURKB	5
MF	GO:0035173	Histone kinase activity	2/12	17/17,697	5.70e−05	0.002	8.40e-04	AURKA/AURKB	2
MF	GO:0008022	Protein C-terminus binding	3/12	187/17,697	2.38e−04	0.004	0.002	CDC20/MAD2L1/TOP2A	3
MF	GO:0004712	Protein serine/threonine/tyrosine kinase activity	2/12	43/17,697	3.75e−04	0.004	0.002	AURKA/AURKB	2
KEGG	hsa04914	Progesterone-mediated oocyte maturation	5/8	100/8076	1.43e−08	2.43e−07	1.51e−07	BUB1/CCNA2/MAD2L1/AURKA/CCNB2	5
KEGG	hsa04110	Cell cycle	5/8	124/8076	4.25e−08	2.94e−07	1.82e−07	BUB1/CCNA2/CDC20/MAD2L1/CCNB2	5
KEGG	hsa04114	Oocyte meiosis	5/8	129/8076	5.19e−08	2.94e−07	1.82e−07	BUB1/CDC20/MAD2L1/AURKA/CCNB2	5

### Characteristic differences of immune microenvironments between FAM-related subtypes

**Figure 4:**
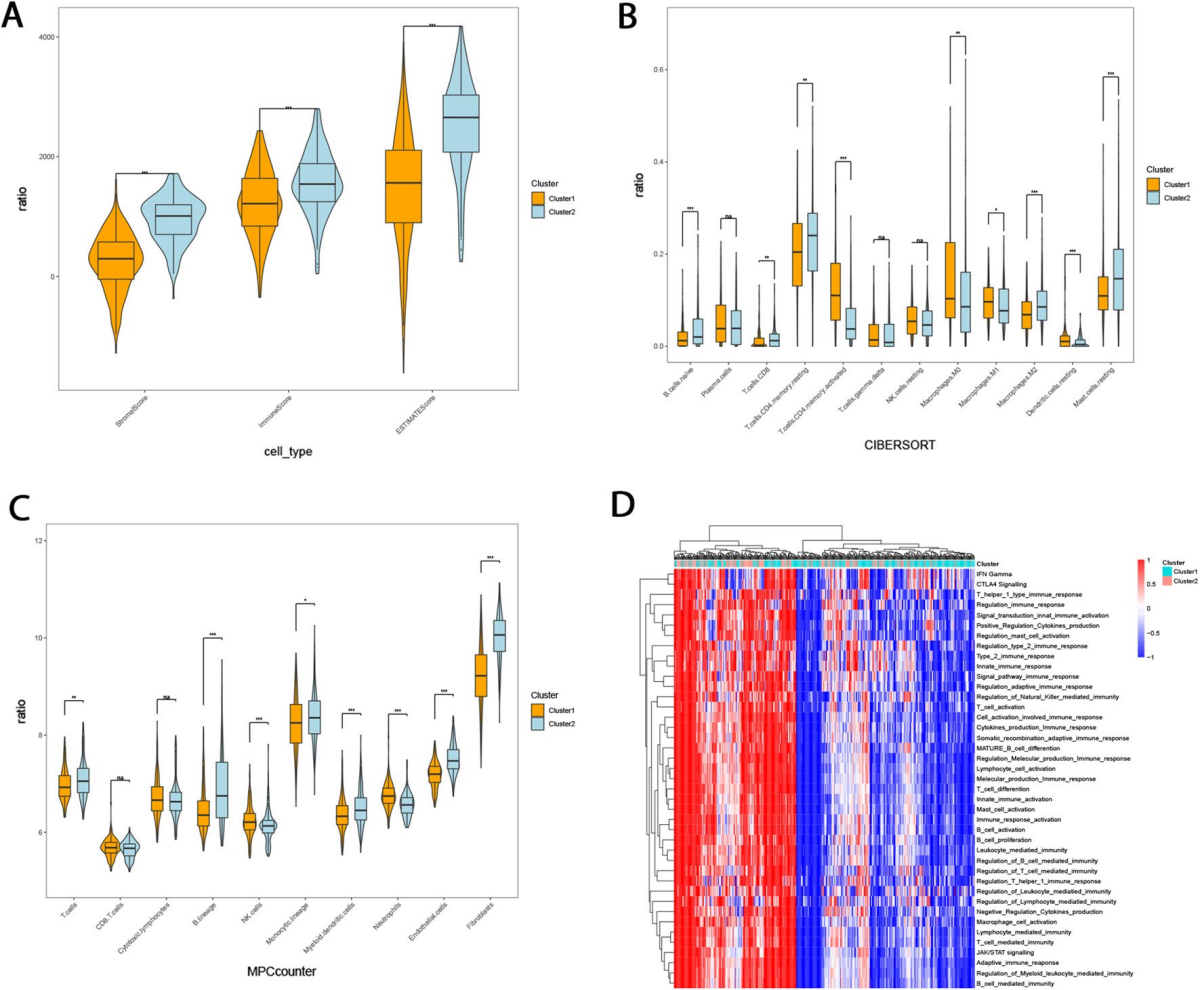
Characteristic differences of immune microenvironments between FAM-related subtypes. (**A**) Tumor purity, immune and stromal scores of FAM subtypes were assessed using an ESTIMATE algorithm. (**B**) Analysis of immune cell infiltration of FAM subtypes using the CIBERSORT algorithm. (**C**) Analysis of immune cell infiltration of FAM subtypes using the MPCcounter algorithm. (**D**) Enrichment analysis of immune-related pathways in FAM subtypes by GSVA algorithm.

Cibersoft and MPCcounter were used to evaluate the immune cell infiltration between different subtypes. Tumor purity, immune, and stromal scores differences between subtypes were assessed using an ESTIMATE algorithm; GSVA was used to determine the enrichment of immune-related pathways between subtypes. The results showed that the stroma score, immune score, and estimation score of Cluster-2 were significantly higher than those of Cluster-1 (Fig. 4A). CIBERSORT results showed that the infiltration of natural B cells, CD8+ T cells, M2-type macrophages, and mast cells in Cluster-2 group were higher than that in Cluster-1 group. In contrast, the infiltration of M0 macrophages, M1 macrophages, and resting dendritic cells in the Cluster-1 group was higher than in the Cluser2 group (Fig. 4B).

The results indicated that the Cluster-1 group had a stronger inflammatory response. In comparison, the Cluster-2 group had stronger specific and non-specific immune responses. In the MCPcouunter method, neutrophils in the Cluster-1 group were significantly increased (p < 0.001), indicating a stronger inflammatory response (Fig. 4C). However, T cells, B cells, Monocytic lineage, Myeloid dendritic cells, endothelial cells, and fibroblasts were significantly increased in Cluster-2 group, indicating stronger immune response and more stromal cell infiltration (Fig. 4C). GSVA analysis showed that there were significant differences in the enrichment of immune pathways among GC FAM-related subtypes. More immune pathways were activated in Cluster-2 (Fig. 4D).

### Correlation between FAM subtypes and expression of immune checkpoint genes and immunomodulatory genes

**Figure 5:**
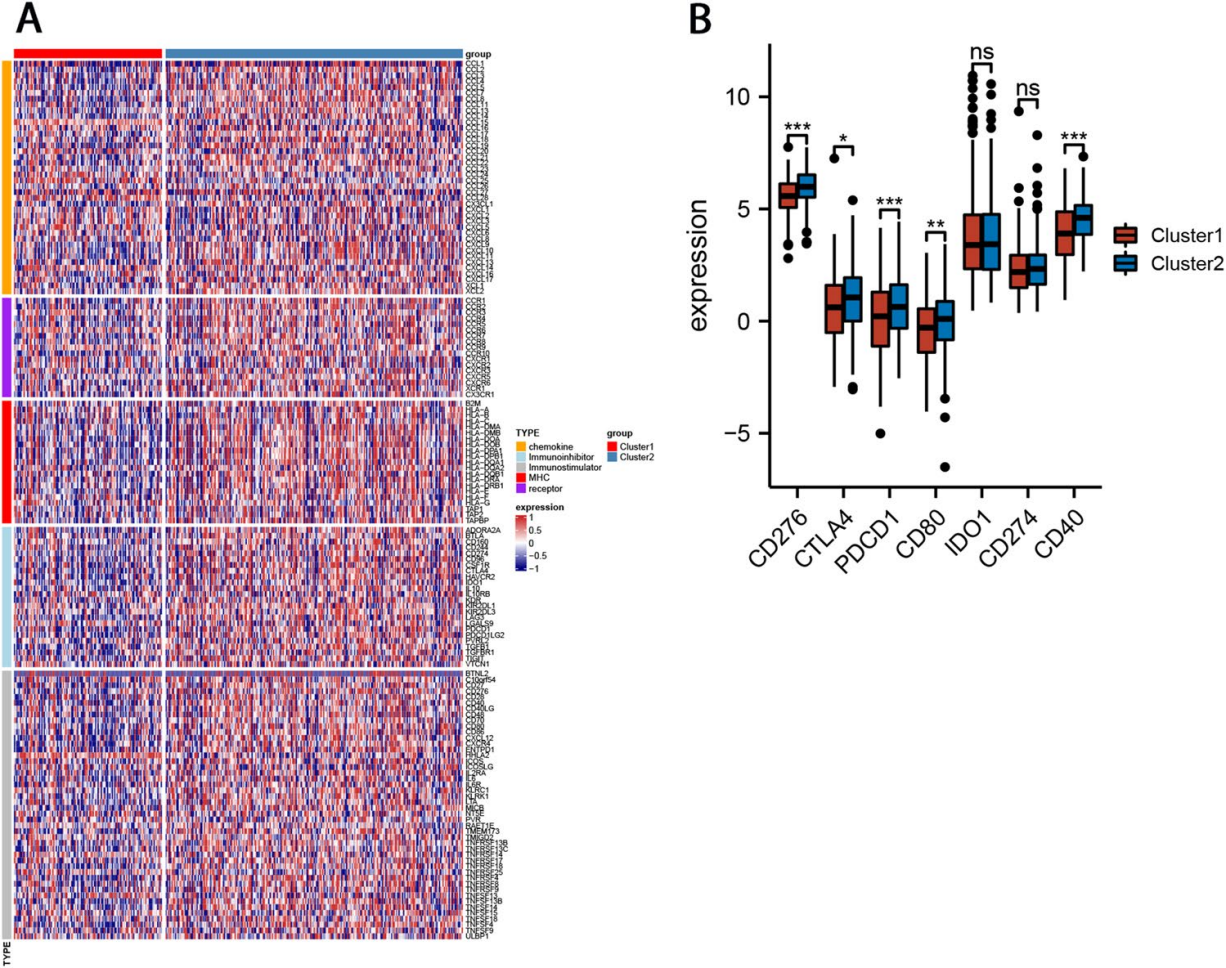
Correlation between FAM subtypes and expression of immune checkpoint genes and immunomodulatory genes. (**A**) The heatmap of the immunoregulatory genes. (**B**) Expression of immune checkpoint genes between two clusters.

To explore whether FAM subtypes of GC are associated with predicting immune checkpoint inhibitor therapy in GC, we analyzed the correlation between the expression of immune checkpoint genes and immunomodulatory genes among different subtypes. Results showed that almost all immune checkpoint genes were upregulated in Cluster-2 GC patients. The differences between CD276, CD40, CD80, CTLA4, and PDCD1 were statistically significant (Fig. 5B). Cluster analysis of immunoregulatory genes, including Chemokine, Immunoinhibitor, Immunostimulator, MHC, and Receptor showed that the expression of immunoregulatory genes was significantly upregulated in Cluster-2 compared with Cluster-1 (Fig. 5A).

## Discussion

The therapeutic effect of GC has been enhanced to some extent with the introduction of minimally invasive techniques, concepts, related instruments, and immunotherapy drugs. Nevertheless, the overall therapeutic outcome remains unsatisfactory. Specific GC populations, such as M SI–H and EBV-positive individuals, can benefit from PD-1 antibodies. Nonetheless, this cohort represents approximately 7% to 8% of the entire population with advanced GC, and there is no clear and effective screening tool for most of the remaining population. Tumor microenvironment (TME) refers to the microenvironment around tumor cells, mainly composed of stromal cells, immune cells, blood vessels, and various signaling molecules. It is usually in a state of immune tolerance^20^.

Immune cells in TME can undergo metabolic reprogramming under the regulation of tumor cells and other signaling molecules, as well as nutrients, to obtain special metabolic characteristics and affect their survival and effector function, thus reducing the efficacy of various immunotherapies such as ICBand tumor vaccine^21–23^. FAM is a key metabolic pathway that regulates immune response, providing energy for immune cells and substrates and precursors for synthesizing cell components and signal molecules^7,24^. Consequently, employing prognostic FAM-related genes, we conducted comparable clustering categorization of GC samples using bioinformatics analysis. Two clusters of GC samples were identified, and it was discovered that each cluster had distinct immunological states, clinicopathological characteristics, and substantial predictive differences. This may, to a certain extent, predict the effectiveness of immunotherapy for GC.

More and more studies have shown that abnormal FAM is involved in the occurrence and development of various cancers, including lung cancer^25^, prostate cancer^26^, colorectal cancer^27^, bladder cancer^28^, etc. FAM-related genes signature is associated with the prognosis of various cancers^29–31^. We first downloaded gene sets related to FAM from the MSigDB database and Genecards database. GSE84437 data set was used to screen out prognostic FAM-related gene sets, including 50 FAM-related genes. GC patients can be divided into 2 groups by the non-negative matrix consensus clustering algorithm and consensus clustering matrix surface. The survival curve showed that Cluster-1 patients had significantly longer overall survival than Cluster-2 patients. Using this gene collection, PCA dimension reduction analysis may efficiently differentiate between the two subtypes of GC patients. Univariate and multivariate Cox regression analyses revealed that FAM-related subtypes were independent predictive variables for patients with GC. We used TCGA-STAD cohort data for validation to test the gene clustering results' stability. The results showed that the TCGA-STAD cohort was still clustered into two groups by non-negative matrix consensus, and the survival curve showed that Cluster-1 patients had significantly longer overall survival than Cluster-2 patients. Changes in energy metabolism in cancer cells compared with normal cells are a new hallmark of most cancers^32^. The results of our study show that variations in FAM may be correlated with the prognosis of GC patients, although the particular mechanism needs to be investigated further.

Furthermore, we explored the clinicopathological features of the two subtypes. Cluster-1 patients were older, with more intestinal GC and earlier T stages. However, there were no significant differences between the two groups in gender, pathological stage, presence or absence of lymph node metastasis, distant metastasis, and anatomical tumor site. In addition, the distribution of the four types of GC molecular subtypes^19^ reported by TCGA was also different between the two groups. MSI molecular subtypes were mainly distributed in Cluster-1 GC patients, while GS molecular subtypes were primarily distributed in Cluster-2 GC patients. These findings demonstrate that the expression status of genes involved in FAM is regulated by patient age, tumor T stage, and tumor Lauren type and is connected with microsatellite instability. Developing the schedule for combination treatments and identifying new cancer therapy targets may benefit from a better understanding the metabolic requirements during the cell cycle.

To explore the internal mechanism of the differences between the two clusters, we analyzed the gene differential expression between the two clusters. The threshold of LogFoldchange and adjusted *P* values were set to 1 and 0.05, and 276 differential genes were obtained, most of which were highly expressed in Cluster-2. Cytoscape software MCODE plug-in was used to screen out the hub modules (threshold set MCODE score ≥ 4, node ≥ 6). The hub module contains 12 genes, which are AURKA, AURKB, CCNA2, CCNB2, CDC20, CDCA3, TOP2A, MAD2L1, CENPA, TPX2, BUB1 and UBE2C. These genes have been reported to promote proliferation and metastasis in multiple tumors^33–39^. Hub genes' functional enrichment study also indicated that these genes were associated with the cell cycle and mitosis. This outcome is also in agreement with the poor prognosis for Cluster-2 patients.

Fatty acids are energy storage and signaling molecules that control defensive mechanisms and developmental processes. They also supply crucial building blocks for the development of membranes^40^. Previous research suggested cell cycle arrest might occur if fatty acid synthesis (FAS) was inhibited. The acetyl-CoA carboxylase (cut6) and fatty acid synthetase (lsd1) mutants have reduced intracellular fatty acid levels, affecting the nucleus and cell division^41^.

Cibersoft and MPCcounter were used to evaluate the immune cell infiltration between different subtypes to explore the effect of FAM on the immune function of GC. Tumor purity, immune, and stromal scores differences between subtypes were assessed using an ESTIMATE algorithm; GSVA was used to evaluate the enrichment of immune-related pathways between subtypes. Our study showed that the tumor immune microenvironment significantly differed between the two subtypes. The infiltration degree of specific and non-specific immune response cells in Cluster-2 was increased considerably, and the immune pathway was greatly enriched, indicating stronger specific and non-specific immune responses in Cluster-2.

Such results demonstrate that FAM-related genomes may be associated with tumor immune microenvironment. FAM has also been found to influence the function of several immune cells in the tumor microenvironment, including T cells and macrophages. In addition, we explored the expression of immune checkpoint (IC) related genes and immune regulation between the two subtypes. In Cluster-2, we found relatively high expression of IC-related genes and immunoregulatory genes. FAM-related genes are associated with biomarkers of the immune checkpoint, which may play an important role in GC immunotherapy. Previous studies have also shown that since PD-1 inhibitor does not affect the metabolic phenotype of tumor-infiltrating lymphocytes^42^, the combination of metabolic reprogramming drugs and PD-1 inhibitor can achieve additive effects. The membrane receptor CD36 targeting fatty acid uptake can inhibit fatty acid uptake by Treg cells, down-regulate fatty acid oxidation of Treg cells, and reduce the number and function of Treg cells ^43,44^ . Targeted CD36 has been shown to act synergistically with PD-1 inhibitors in mouse models^45^. According to Cui et al., aberrant FAM impacted GC growth. Chemotherapy drug resistance and recurrence were linked to the abnormal expression of FAM-related genes^46^. Sterol O-acyltransferase (SOAT)1 was highly expressed in GC tissues and negatively correlated with GC prognosis via regulation of SREBP-1 and SREBP-2 expression. SREBP-1 and SREBP-2 increased the expression of vascular endothelial growth factor C (VEGF-C), which promoted lymphangiogenesis^47^.

Additionally, FAM is crucial for the malignant proliferation of tumors through invasion and metastasis. For instance, stimulation of the TGFβ signaling pathway in lung cancer cells decreases fatty acid production by inhibiting ChREBP, and knockdown of FASN decreases E-cadherin expression, which increases lung cancer cell invasion and metastasis^48^. Since only a few investigations have determined the role of FAM in GC, possible novel FAM pathways in GC require further exploration.

## Conclusions

In conclusion, this study classified GC samples into two clusters by NMF consensus clustering using the FAM genes associated with prognosis, and there were significant prognostic differences between those two clusters. FAM-related subtypes are independent prognostic risk factors in GC patients. FAM-related subtypes have potential correlations with the immune microenvironment of GC. They are significantly correlated with biomarkers of immune checkpoints, which may play an important role in GC immunotherapy. In the next step, we will conduct cell biology experiments and clinical verification of our findings.

## Methods and materials

### Data sets and FAM genes

From Gene Expression Omnibus (GEO) database (https://www.ncbi.nlm.nih.gov/geo/query/acc.cgi?acc=GSE84437) to download GSE84437 datasets, which was a microarray data of mRNA expression in GC, and corresponding clinicopathological and prognostic information, as a training dataset. From The Cancer Genome Atlas (TCGA) data portal (https://gdcportal.nci.nih.gov/) to download the RNA sequencing data and corresponding clinicopathological and prognostic information of TCGA-STAD cohort as a validation dataset. From the Molecular Signature Database v.7.4 (MSigDB; https://www.gsea-msigdb.org/gsea/msigdb), three FAM gene sets (REACTOME_FATTY_ACID_METABOLISM, KEGG_FATTY_ACID_METABOLISM, HALLMARK_FATTY_ACID_METABOLISM) were downloaded. The keyword "fatty acid metabolism" was searched in the Genecards database, and genes with a score greater than 50 were regarded as Genecards FAM gene sets. A total of 332 genes related to FAM were obtained after removing duplicate genes (Supplementary Table S1).

### Identification of prognostic FAM genes

Survival R package and Cox univariate regression analysis were used to analyze the training set, and the predictive FAM genes were screened out. A false discovery rate (FDR) < 0.05 was set as the cutoff criterion.

### Identification and predictive analysis of FAM gene subtypes in GC

The "NMF" R package^49^ was used for unsupervised non-negative matrix consensus clustering of the normalized expression data of the prognostic FAM gene set. For each cluster number (k in 2:10), the NMF was run 50 times to evaluate cluster stability. The optimal cluster number k = 2 was selected for the magnitude of the cophenetic coefficient began to decrease. The "PCA" R package was used for dimension reduction analysis, the Survival "R package for subtype predictive analysis, and P < 0.05 was used as the significant difference threshold.

### Differential expression gene analysis, protein–protein interaction analysis, and functional enrichment analysis of subtypes

Differential gene expression between subtypes was analyzed using the "Limma" R package^50^. The adjusted P values for multiple tests were calculated using Benjamini-Hochberg. The absolute value of logFC greater than 1 and FDR < 0.05 were regarded as the threshold of significantly differentially expressed genes. PPI analysis was performed on the differential gene set by the String database, and the MCODE plug-in of Cytoscape software screened the hub module. The threshold was set as MCODE score ≥ 4 and node ≥ 6. The "clusterProfiler" R package^51^ was used for GO and KEGG^52^ enrichment analysis of differentially expressed genes, with an adjusted p value less than 0.05 as the threshold of significant enrichment. The "ggplot2" R package^53^ was used to visualize the results.

### Immune infiltration analysis

The CIBERSORT (https://cibersort.stanford.edu/) and MPCcounter platforms were utilized to evaluate the immune cell infiltration of GC patients. Only the samples with p < 0.05 were included in subsequent immune infiltration analysis in CIBERSORT. The ESTIMATE package was used to calculate the immune score, stromal score, and tumor purity of GC.

### Statistical analysis

R software (https://www.r-project.org/) was used for all calculations and statistical analysis. Unpaired Student's T test or Mann Whitney U test were used to compare two groups of variables with normal or non-normal distribution. The chi-square test or Fisher exact test was used for categorical data. Survival analysis was performed using the "survival" R package. Log-rank test was used to determine whether there was a significant difference in survival curves.

### Supplementary Information

Supplementary Figure S1.

Supplementary Figure S2.

Supplementary Figure S3.

Supplementary Figure S4.

Supplementary Figure S5.

Supplementary Figure S6.

Supplementary Figure S7.

Supplementary Table S1.

Supplementary Table S2.

Supplementary Table S3.

## Data Availability

The datasets ANALYZED for this study can be found in The Cancer Genome Atlas (TCGA, https://portal.gdc.cancer.gov/) and Gene Expression Omnibus (GEO) database (https://www.ncbi.nlm.nih.gov/geo/query/acc.cgi?acc=GSE84437).
