# Supplementary figures and images for: Identification and immune landscape analysis of fatty acid metabolism genes related subtypes of gastric cancer
Source: Sci Rep. 2023 Nov 22;13:20443. doi: 10.1038/s41598-023-47631-6 (PMC10665388; doi:10.1038/s41598-023-47631-6)

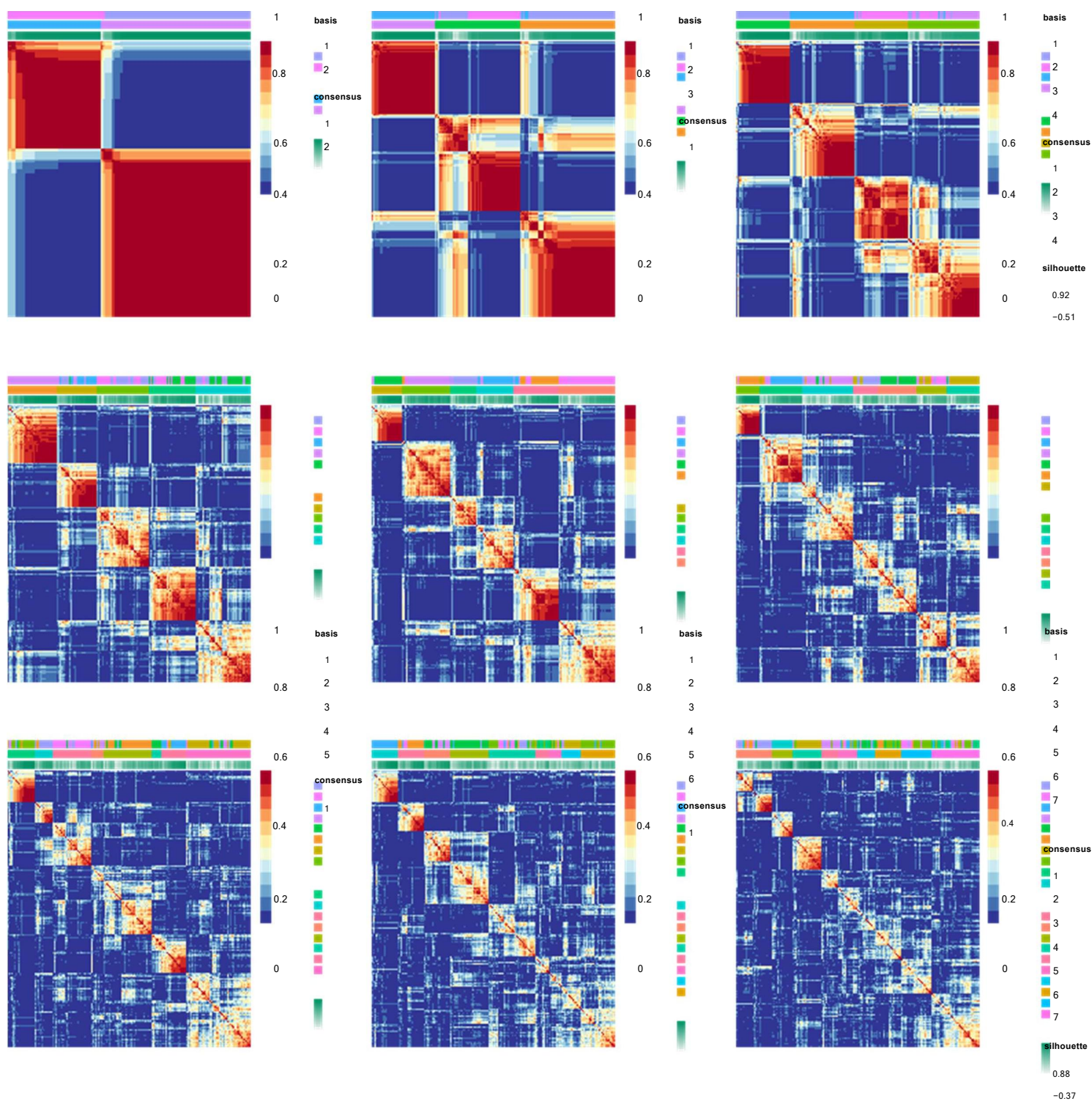

Figure S1. The consensus map of NMF clustering results in the GSE84437 dataset with rank  $k$  from 2 to 10.

Supplement: Supplementary file 1 — Supplementary Figure S1. [file 41598_2023_47631_MOESM1_ESM.pdf]

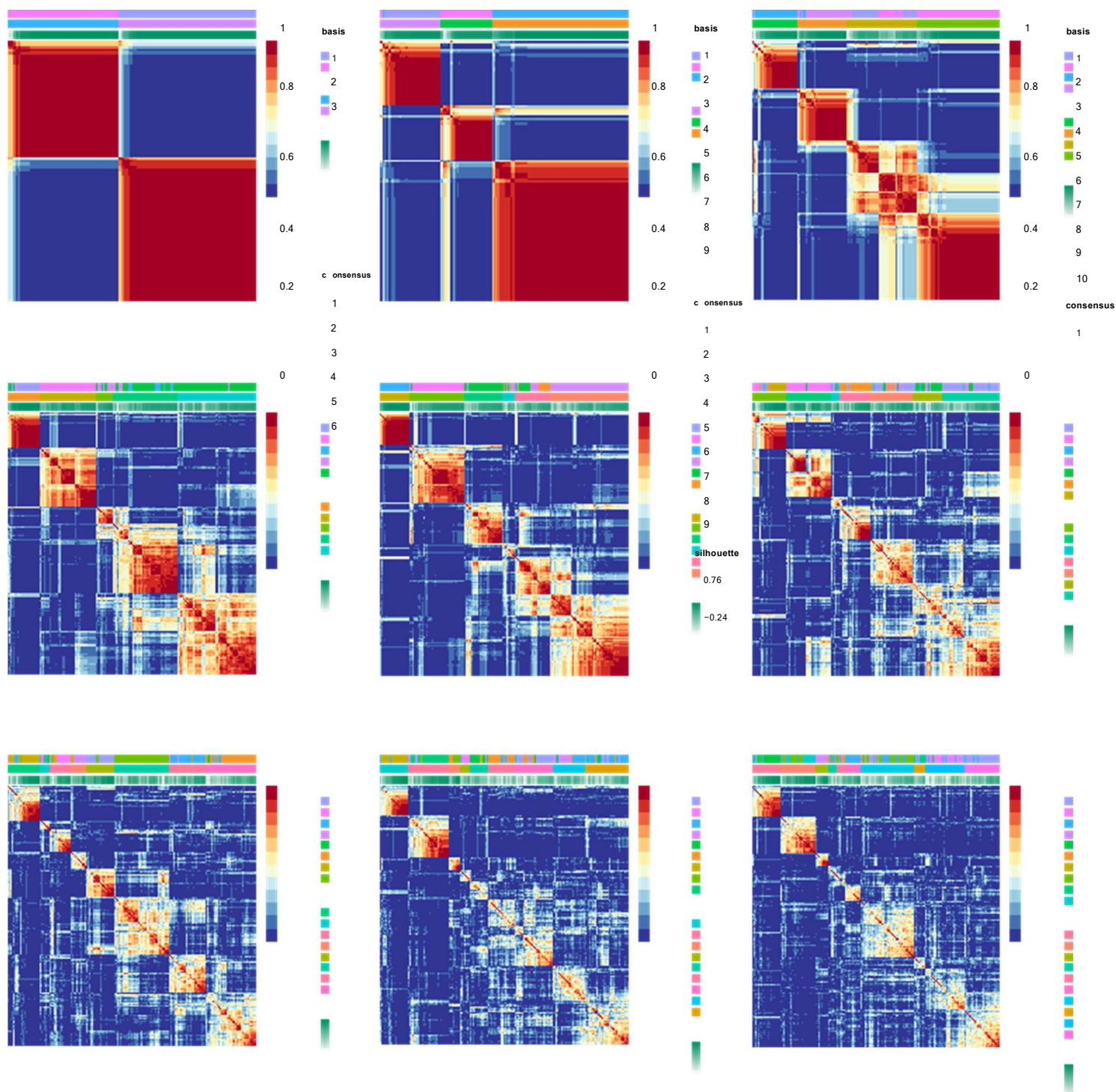

Figure S2. The consensus map of NMF clustering results in the TCGA-STAD dataset with rank  $k$  from 2 to 10.

Supplement: Supplementary file 2 — Supplementary Figure S2. [file 41598_2023_47631_MOESM2_ESM.pdf]

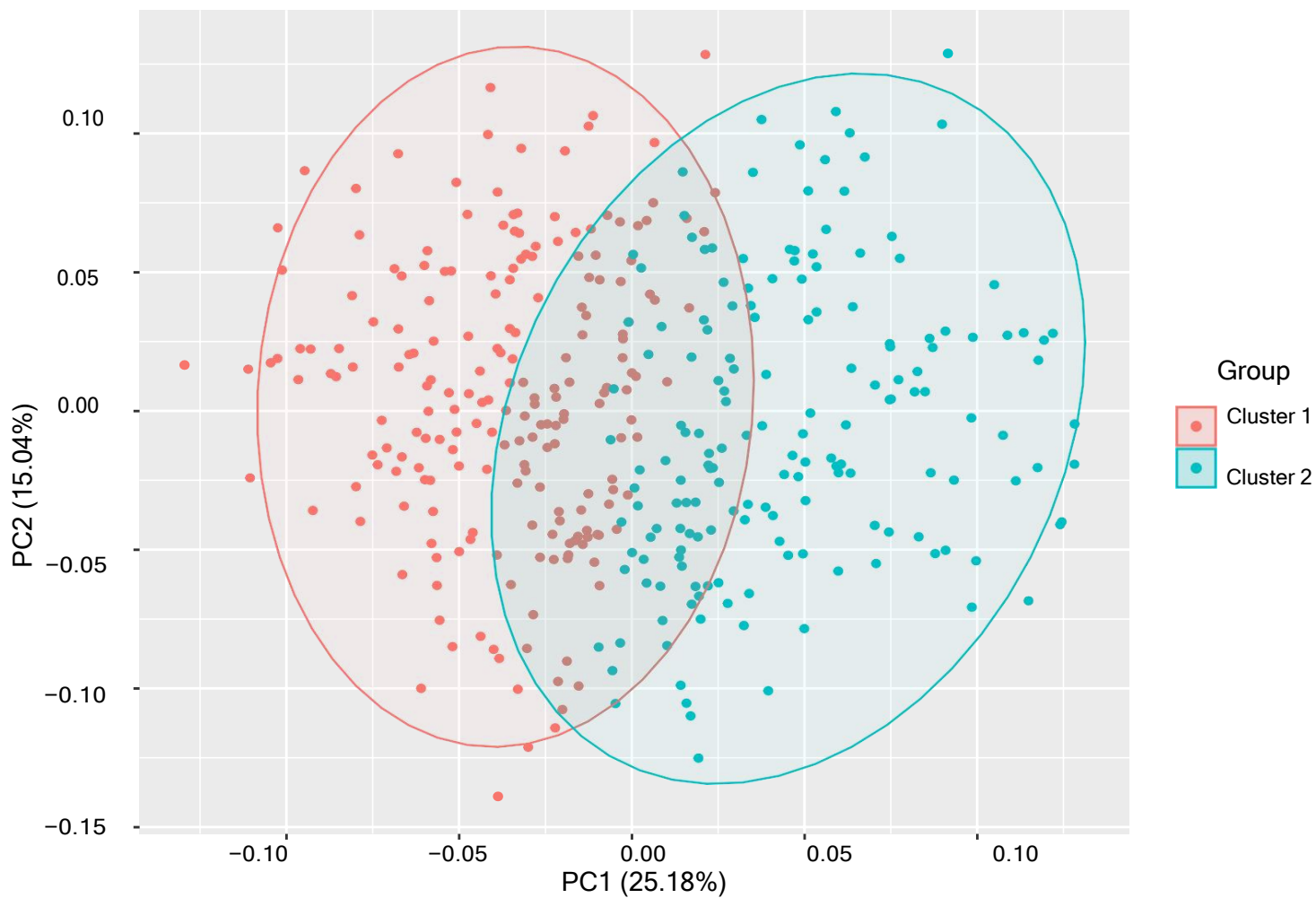

Figure S4: The principal component analysis results for TCGA-STAD cohort samples

Supplement: Supplementary file 4 — Supplementary Figure S4. [file 41598_2023_47631_MOESM4_ESM.pdf]

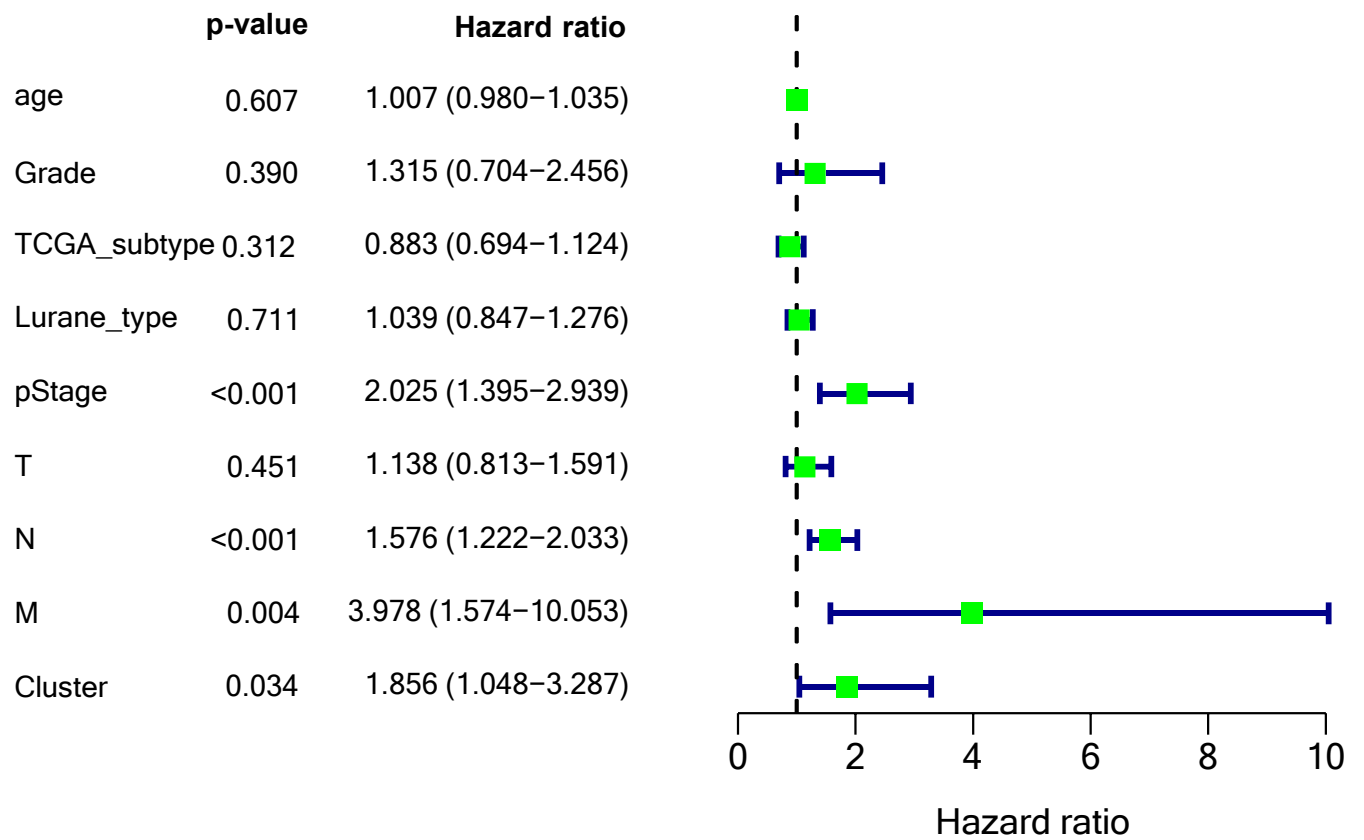

Figure S6. Forest plot of TCGA-STAD cohort univariable Cox regression analysis.

Supplement: Supplementary file 6 — Supplementary Figure S6. [file 41598_2023_47631_MOESM6_ESM.pdf]

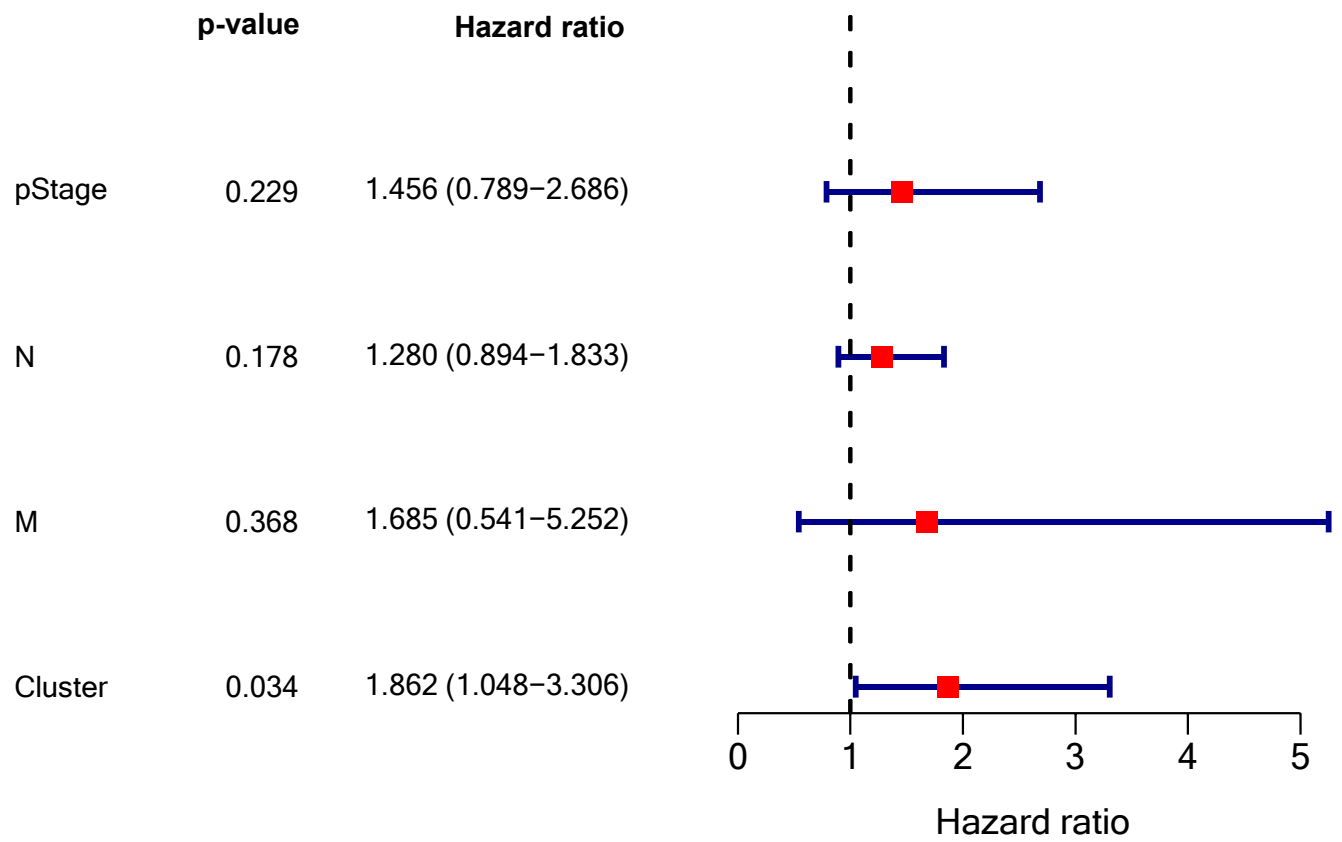

Figure S7. Forest plot of TCGA-STAD cohort multivariable Cox regression analysis.

Supplement: Supplementary file 7 — Supplementary Figure S7. [file 41598_2023_47631_MOESM7_ESM.pdf]
