# Supplementary material for: Identification and immune landscape analysis of fatty acid metabolism genes related subtypes of gastric cancer
Source: Sci Rep. 2023 Nov 22;13:20443. doi: 10.1038/s41598-023-47631-6 (PMC10665388; doi:10.1038/s41598-023-47631-6)
Supplement: Supplementary file 3 — Supplementary Figure S3. [file 41598_2023_47631_MOESM3_ESM.pdf]

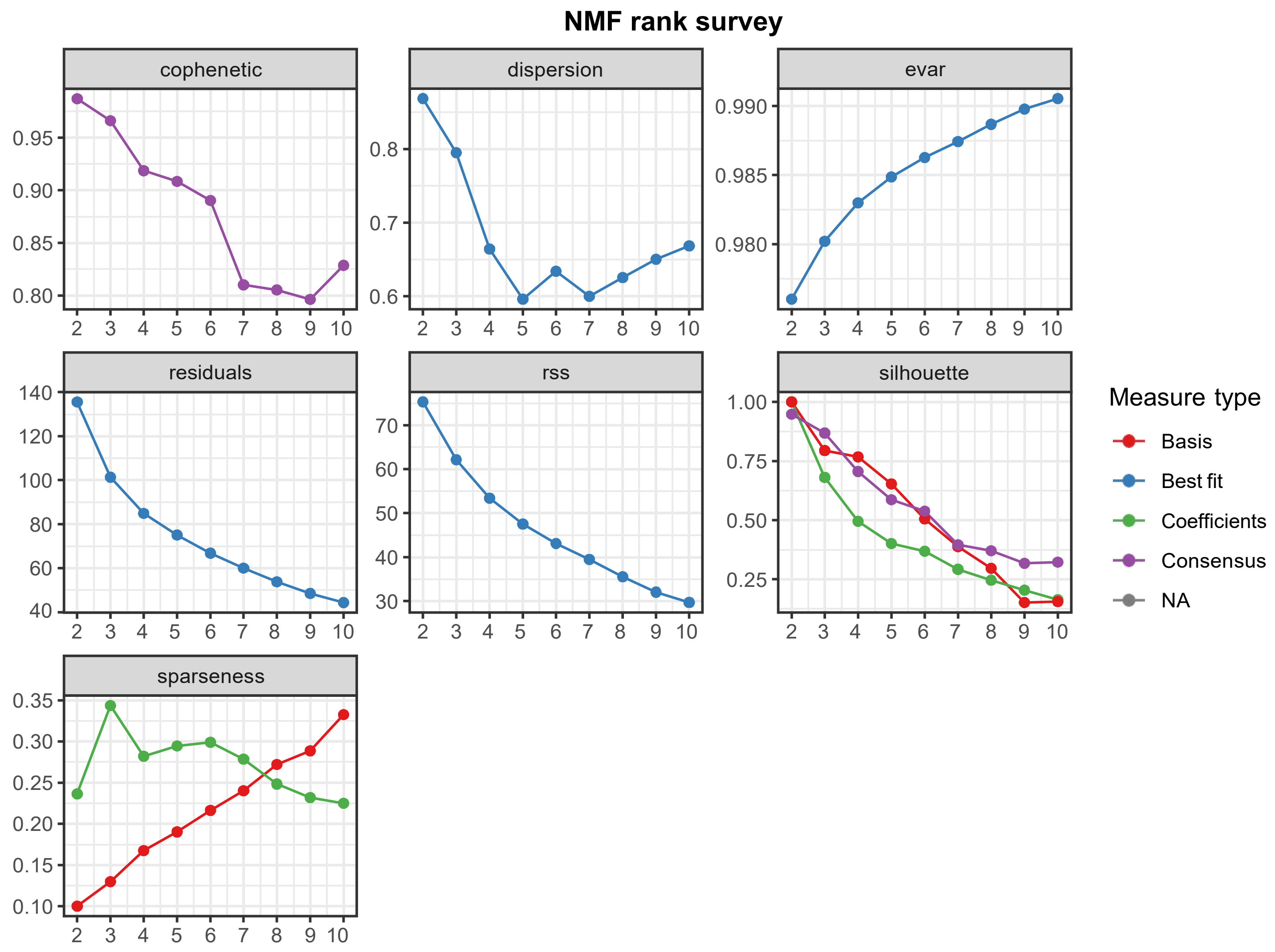

Figure S3. The trend of the cophenetic, dispersion, evar, residuals, rss, silhouette and sparseness coefficients at different rank k.
