# Supplementary material for: Identification and immune landscape analysis of fatty acid metabolism genes related subtypes of gastric cancer
Source: Sci Rep. 2023 Nov 22;13:20443. doi: 10.1038/s41598-023-47631-6 (PMC10665388; doi:10.1038/s41598-023-47631-6)
Supplement: Supplementary file 5 — Supplementary Figure S5. [file 41598_2023_47631_MOESM5_ESM.pdf]

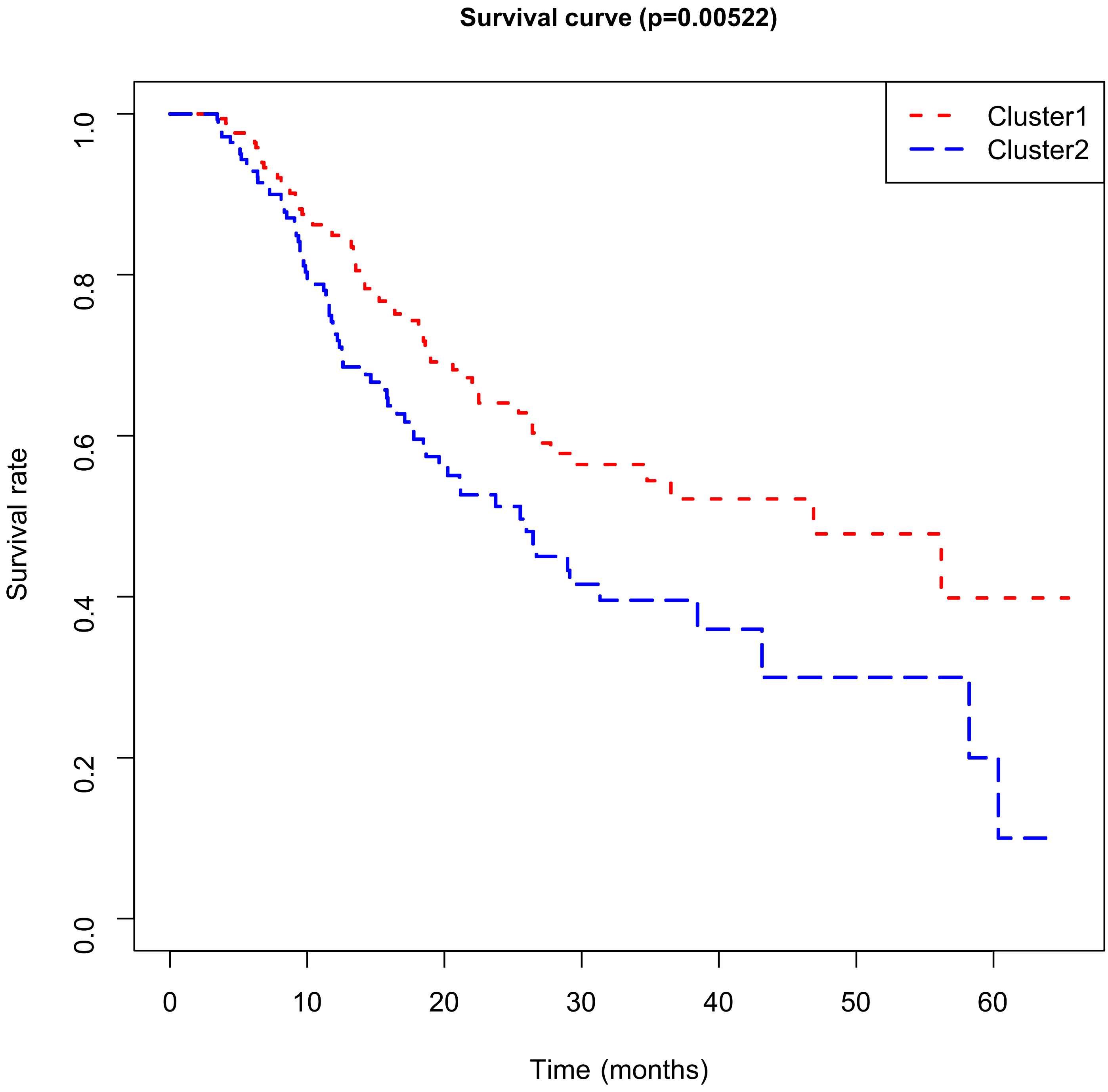

Figure S5. Kaplan-meier survival curves of overall survival in Cluster1 and Cluster2 gastriccancer patients.
